# High-throughput homogenous assay for the direct detection of *Listeria monocytogenes* DNA

**DOI:** 10.1038/s41598-024-56911-8

**Published:** 2024-03-25

**Authors:** Cheryl M. Armstrong, Joseph A. Capobianco, Sarah Nguyen, Manita Guragain, Yanhong Liu

**Affiliations:** United States Department of Agriculture, Agricultural Research Service, Eastern Regional Research Center, 600 East Mermaid Ln., Wyndmoor, PA 19038 USA

**Keywords:** Biological techniques, Molecular biology

## Abstract

The Amplified Luminescent Proximity Homogenous Assay-linked Immunosorbent Assay (AlphaLISA) is known for detecting various protein targets; however, its ability to detect nucleic acid sequences is not well established. Here, the capabilities of the AlphaLISA technology were expanded to include direct detection of DNA (aka: oligo-Alpha) and was applied to the detection of *Listeria monocytogenes.* Parameters were defined that allowed the newly developed oligo-Alpha to differentiate *L. monocytogenes* from other *Listeria* species through the use of only a single nucleotide polymorphism within the 16S rDNA region. Investigations into the applicability of this assay with different matrices demonstrated its utility in both milk and juice. One remarkable feature of the oligo-Alpha is that greater sensitivity could be achieved through the use of multiple acceptor oligos compared to only a single acceptor oligo, even when only a single donor oligo was employed. Additional acceptor oligos were easily incorporated into the assay and a tenfold change in the detection limit was readily achieved, with detection limits of 250 attomole of target being recorded. In summary, replacement of antibodies with oligonucleotides allows us to take advantage of genotypic difference(s), which both expands its repertoire of biological markers and furthers its use as a diagnostic tool.

## Introduction

Enzyme-linked immunosorbent assays (ELISAs) have a long history of use as diagnostic tools in both the medical and agricultural fields for the identification and quantification of targets of interest^1^. These plate-based assays are typically constructed by immobilizing capture molecules specific to the target of interest onto the surface of a 96 or 384-well polystyrene plate. Sample material is then added into the wells of the plate, which allows binding of the target to the capture molecule. Detection of the target can be subsequently achieved using a second recognition element such as an enzyme-conjugated antibody specific for the target of interest. Through the addition of enzyme substrate, measurable signals are generated via the activity of the enzymes that are conjugated to the antibodies. Target immobilization on the plate enhances the assay by allowing unbound material to be removed prior to detection via washing of the plate wells, which not only helps minimize reaction volumes but also permits analyte measurement from crude sample preparations. Although critical for the procedure, washing requires both time and additional manipulation of the assay plates. Therefore, a “no wash” alternative to the ELISA was devised.

This “no wash” alternative is known as the AlphaLISA, or the Amplified Luminescent Proximity Homogenous Assay-linked Immunosorbant Assay. The basis for the system relies on the ability of two different antibody-coated beads to reside within a 200 nm distance of one another when bound to a target of interest within the solution^2^. One bead, known as the donor, contains a photosensitizer that has been shown to emit singlet oxygen molecules into solution upon excitation with a laser. The second bead, known as the acceptor, then reacts with the singlet oxygen molecules that were produced and, through a cascade of events, produces a fluorescent signal measurable at 615 nm. The distance between the beads is limited to 200 nm because this denotes the maximum migration distance that can be traveled by a singlet oxygen molecule prior to decay back to its ground state where it no longer contains sufficient energy to activate the acceptor bead complex. The design of the AlphaLISA can be advantageous to high-throughput testing laboratories because of its simpler protocol (where all reagents are simply overlaid upon one another and wash steps are eliminated), which both reduces assay time and helps facilitate automation^3^. However, wash steps within an assay aid in the removal of unwanted components. Therefore, the lack of wash steps creates additional design constraints for the AlphaLISA compared to the ELISA.

The overall ease of use and rapid response times of both the ELISA and AlphaLISA has led to a desire to expand the assay’s capabilities from the quantitative determination of protein antigens to the detection of other biomolecules such as nucleic acids. Methods based upon nucleic acids can be vital, especially when antibodies for identification are not readily available. To allow for detection of nucleic acids, the antibodies and antigenic ligands have been replaced with oligonucleotides that can capture complementary nucleic acid analytes, which are ultimately detected by probes. This has been widely achieved with the ELISA, especially when used in combination with the polymerase chain reaction (a technique commonly known as PCR-ELISA)^4–7^. Conversely, it has not been reported for the AlphaLISA and has only been reported using a similar bead-based type of technology referred to as AlphaScreen^8^. However, detection in that assay was performed indirectly, requiring both bead-conjugated oligos and bridging detection oligos to allow the beads to bind instead of directly binding the DNA as presented here.

*Listeria* are Gram-positive, facultative anaerobic bacteria, that can be found in many different environments^9^. The genus is currently subdivided into > 20 identified species^10^. *Listeria monocytogenes* is a known human pathogen that can be present in ready-to-eat foods and has been the reason for several food safety recalls within the United States^11^. Fatality rates are often higher for *L. monocytogenes* compared to other common foodborne diseases. For example, multiple *Listeria* outbreaks have been reported in recent years including a multistate outbreak in 2021 associated with packaged salads where 16 of the 18 people affected were hospitalized and three deaths reported, and a deli meat and cheese outbreak in 2022 where 13 of the 16 people infected were hospitalized and one death was reported^12^. Because of the severity of the infections, detection and removal of sources contaminated with *L. monocytogenes* is a critical aspect in food safety^13^. Unfortunately, immunological based detection methods for *L. monocytogenes* have been reported to have low specificity for reasons such as high genetic diversity amongst strains, inconsistent antigen expression, and the presence of the antigenic targets on other *Listeria* species and/or non-*Listeria* targets^14^. This suggests that non-antibody-based methods for detection may be the most appropriate.

Given that differentiation of *L. monocytogenes* from other *Listeria* species is important in the context of foodborne pathogens since only *L. monocytogenes* is consistently associated with human illness and the low specificity of the current immunological-based detection methods for *L. monocytogenes*, we have developed an oligo-Alpha for the direct detection of nucleic acids from *L. monocytogenes* by employing oligonucleotides for the 16S rDNA region and sequence-specific capture methods. We have also investigated several parameters that constrain the design of the assay and provide insight into methods to achieve greater sensitivity. Applications for this oligo-Alpha go beyond detection of *L. monocytogenes* since a simple alteration to the oligonucleotides employed can result in extending the technology to the detection of other organisms.

## Materials and methods

### Bacterial strains, genomic DNA isolation, and amplification

The *Listeria* strains used in this study (Supplemental Table 1), kept in glycerol stocks at − 80 °C, were streaked onto plates of Brain Heart Infusion (BHI) (BD Difco, Franklin Lakes, NJ) and incubated at 37 °C overnight. Single colonies from each strain were picked from the BHI plates and used to inoculate 5 mL of BHI broth in a 15 mL snap cap tube. Tubes were subsequently incubated at 37 °C for 18 h undergoing agitation at 175 RPM. Genomic DNA was isolated from the individual strains using the Qiagen DNeasy® Blood Tissue kit (Qiagen, Valencia, CA, USA) according to the manufacturer's instructions for Gram positive bacteria. Fragments of *Listeria* 16S rDNA were subsequently amplified from the extracted genomic DNA using the Qiagen Multiplex PCR kit and primers U1 and LI1 (Integrated DNA Technologies, Coralville, IA) (Supplemental Table 2). Each amplification reaction contained a final concentration of the 1 × solution of the QIAGEN Multiplex PCR Master Mix, 0.31 µM of each U1 and LI1 primer, ~ 0.5 µg genomic DNA as a template and RNase-free water to bring the total reaction volume to 50 µL. The PCR parameters used for the amplification from all *Listeria* spp. were performed on the Bio-Rad T100™ Thermal Cycler using conditions identical to those previously reported^15^ with the ~ 938 bp fragments being purified post amplification using the QIAQuick PCR Purification Kit (Qiagen). The concentration of all genomic DNA and amplified fragments were determined via the DS-11 + spectrophotometer (DeNovix Inc., Wilmington, DE).

### Optimization of binding conditions

Binding conditions were optimized by varying the concentration of MgCl_2_ and KCl utilized in the buffer. Initially, six different MgCl_2_ concentrations were tested (0, 1 mM, 2 mM, 3 mM, 4 mM, and 5 mM) in a 1 × Mg^−^ assay buffer consisting of 10 mM Tris–Cl, 50 mM KCl, and 200 µg/mL bovine serum albumin at pH 8.0. These buffers were used for the dilution of the oligos, donor beads, acceptor beads and DNA subsequently added to the final assay as described. A stock solution of the 16S DNA fragment amplified from *L. monocytogenes* was diluted to 36 ng/µL, heated to 95 °C for 10 min, and then cooled to 4℃ to separate the complimentary DNA strands. Then, 90 ng of that DNA stock solution was added to a 96-well ½ area grey assay plate (Perkin Elmer, Shelton, CT) along with 3 nM of L. mono_16S-Rev12 to act as the donor oligo, 1 nM of L. mono_16S-Rev7 to act as the acceptor oligo, and the 1X Mg^−^ assay buffer being tested to reach a 12.5 µL total reaction volume. Plates were sealed with TopSeal-A film (Perkin Elmer), spun briefly to settle the contents, and incubated at 50 °C for 30 min. AlphaScreen Streptavidin Alpha Donor Beads (Perkin Elmer 6760002) were diluted to 160 µg/mL while the AlphaLISA Anti-Digoxigenin (DIG) Acceptor Beads (Perkin Elmer AL113C) were diluted to 40 µg/mL using the aforementioned buffer. After the incubation, the film was removed from the plate and 6.25 µL of the diluted AlphaScreen Streptavidin Alpha Donor Beads and 6.25 µL of the diluted AlphaLISA Anti-DIG Acceptor Beads were added, which resulted in a final assay volume of 25 µL. All processing steps that included the beads were performed under limited light conditions. After the addition of both beads, plates were sealed and placed into the Envision Multilabel plate reader (PerkinElmer) to undergo the following operations: (1) The temperature control for the duration of the experiment was set at 50 °C. (2) A shake duration of 30 s using a speed of 60 RPM with a 10 mm diameter inside orbital shake was performed. (3) An incubation of 30 min was performed and (4) the plate was read using the AlphaScreen emission 570 nm wavelength filter with an excitation time of 0.18 s and an emission time of 0.37 s.

The optimal concentration of KCl was subsequently defined by varying the amount of KCl (0, 25 mM, 50 mM, 75 mM, and 100 mM) in a buffer consisting of 10 mM Tris–Cl, 4 mM MgCl_2_ and 200 µg/mL bovine serum albumin at pH 8.0. DNA, in addition to streptavidin donor/anti-DIG acceptor oligos and the Alpha donor/acceptor beads, were added to the assay with the reaction proceeding in the same manner as described above for the MgCl_2_ concentration tests.

### Specificity testing for *L. monocytogenes*

Specificity tests were conducted within 1X assay buffer [10 mM Tris–Cl, 4 mM MgCl_2_, 50 mM KCl, 200 µg/mL bovine serum albumin at pH 8.0] containing L. mono_16S-Rev7 (acceptor oligo), either L. mono_16S-Rev12 or L. mono_16S-Rev13 (donor oligo), both Alpha streptavidin donor and anti-DIG acceptor beads, and 90 ng of *Listeria* DNA. The reaction proceeded in the same manner as described above for the MgCl_2_ concentration optimization. For specificity testing, a ~ 938 bp PCR product of *Listeria* DNA amplified from the following species of *Listeria* were utilized: *L. monocytogenes*, *L. innocua*, *L. welshmeri*, *L. seeligeri*, *L. grayii* and *L. ivanovii* (Supplemental Table 1).

### Defining the limit of detection

Detection limits for the assay were performed as described above for the specificity testing except a tenfold dilution series of *L. monocytogenes*, *L. innocua*, or a combination of *L. monocytogenes* and *L. innocua* DNA was included. Total DNA concentrations were 90, 9, 0.9, 0.09, 0.009, 0.0009, and 0.00009 ng for assays consisting of a single *Listeria* species, whereas DNA concentrations were doubled for assays containing two species; ranging from 180 ng to 180 pg since equal amounts of DNA from each species was included.

### Improving assay sensitivity

Assays were performed using additional acceptor oligos to increase the sensitivity of the assay. Prior to performing this line of investigation, the Design of Experiment (DOE) method was employed to help define the optimal amount of oligos to be used since additional acceptor oligos were being incorporated (data not shown). Once optimal amounts were determined, experiments were then performed to identify if additional anti-DIG acceptor beads should be added to accommodate the increased number of acceptor oligos being utilized (data not shown). Based on these data, it was determined that acceptor oligos should be utilized at 2.5 nM concentrations and that doubling the amount of anti-DIG acceptor beads (from 40 µg/mL to 80 µg/mL) provided increased signal intensities for experiments containing > 1 acceptor oligo.

Subsequent assay performed to determine the effect that additional acceptor oligos had on signal intensities contained 10 µL of oligos (consisting of a mixture of 7.5 nM of L. mono_16S-Rev12 (donor oligo) and 2.5 nM of each acceptor oligo(s) used: L. mono_16S-Rev1, L. mono_16S-Rev5, and/or L. mono_16S-Rev8), 12.5 µL of beads [consisting of both the Alpha streptavidin donor (160 µg/mL) and Anti-DIG acceptor beads (80 µg/mL)], and 2.5 µL of oligo L. mono_16S-Seq (1193–1387) with concentrations varying from 0 to10 nM. (The oligo L. mono_16S-Seq (1193–1387) was used in place of the PCR-amplified 16S rDNA fragment from *L. monocytogenes* as the target to provide accurate and consistent measurements of the amount of DNA within in the assay). The steps of the reaction proceeded in the same manner as described above for the MgCl_2_ concentration tests. Controls contained the donor and various acceptor oligos but did not include any of the L. mono_16S-Seq (1193–1387) target (no DNA) or contained the various concentrations of L. innocua_16S-Seq (1134–1328) in place of the L. mono_16S-Seq (1193–1387) oligo.

### Matrix compatibilities

The ability of the assay to detect *L. monocytogenes* in buffer, apple juice, and non-fat milk was tested. Buffer was identical to the 1X assay buffer used above, apple juice was purchased from a local grocer, and the non-fat milk solution was sourced from a milk powder that was made according to the manufacturer’s recommendation where 1.6 g of non-fat milk was mixed with 11.25 mL of water. The assay contained L. mono_16S-Rev1, L. mono_16S-Rev5, and L. mono_16S-Rev8 as the acceptor oligos, the L. mono_16S-Rev12 as the donor oligo, both Alpha streptavidin donor and anti-DIG acceptor beads, and varying concentrations of oligo L. mono_16S-Seq (1193–1387) in the same fashion as described above. The only difference was that the L. mono_16S-Seq (1193–1387) oligo was diluted from a 100 µM stock in buffer, apple juice, or milk to the target concentrations (0.01, 0.1, 1, 10, and 100 nM) to represent the 16S rDNA fragment from *L. monocytogenes* in those matrices. Controls contained the various matrix agents but did not include any of the L. mono_16S-Seq (1193–1387) target.

### *L. monocytogenes* lysates

Detection of *L. monocytogenes* from lysates using the oligo-Alpha was performed by inoculating 5.5 mL of BHI with a single colony of either *L. monocytogenes* 33435 or *L. innocua* 51742. Cells were grown with shaking at 35 °C for ~ 5 h (OD_600_ between 0.56 and 0.66 for *L. monocytogenes* and 0.78–0.96 for *L. innocua*). A 2 mL aliquot was removed and cells were pelleted via centrifugation at 1500×*g* for 2 min. The pellets were subsequently resuspended in 0.5 mL DNA/RNA shield (Zymo Resesarch, Irvine, CA) and frozen at − 80 °C until use. Cells were lysed using the OmniLyse cell disruptor (Claremont BioSolutions, Upland, CA) following the manufacture’s protocol to ensure lysis of the Gram positive bacteria. Serial dilutions of the lysate were then made in a solution containing 1 part DNA/RNA shield for every 9 parts 1 × TKMB to help prevent degradation of the nucleic acids within the cell lysates. The oligo-Alpha was conducted as described above at 52 °C using the L. mono_16S-Rev1, L. mono_16S-Rev5, and L. mono_16S-Rev8 as the acceptor oligos, the L. mono_16S-Rev12 as the donor oligo, along with the Alpha streptavidin donor and anti-DIG acceptor beads, and the serial diluted *Listeria* lysates. The 6 × 6 drop plate method was performed to enumerate the number of cells within the culture as described previously^16^, except droplets contained only 7 μL of culture.

### Statistical analysis

Data analysis including graphing and statistical processing was carried out using JMP 16.2.0. Comparisons for each pair were performed via a Student’s t-test with the data used to define significance amongst oligo-Alpha signals. An Analysis of Variance (ANOVA) was also performed to define the ability of the oligo-Alpha to detect *L. monocytogenes* in cell lysates, with pairwise comparisons between groups being determined by Tukey’s honestly significant difference (HSD) tests. The level of significance was set at 0.05 for all tests performed.

## Results

### Production of an oligo-Alpha

**Figure 1:**
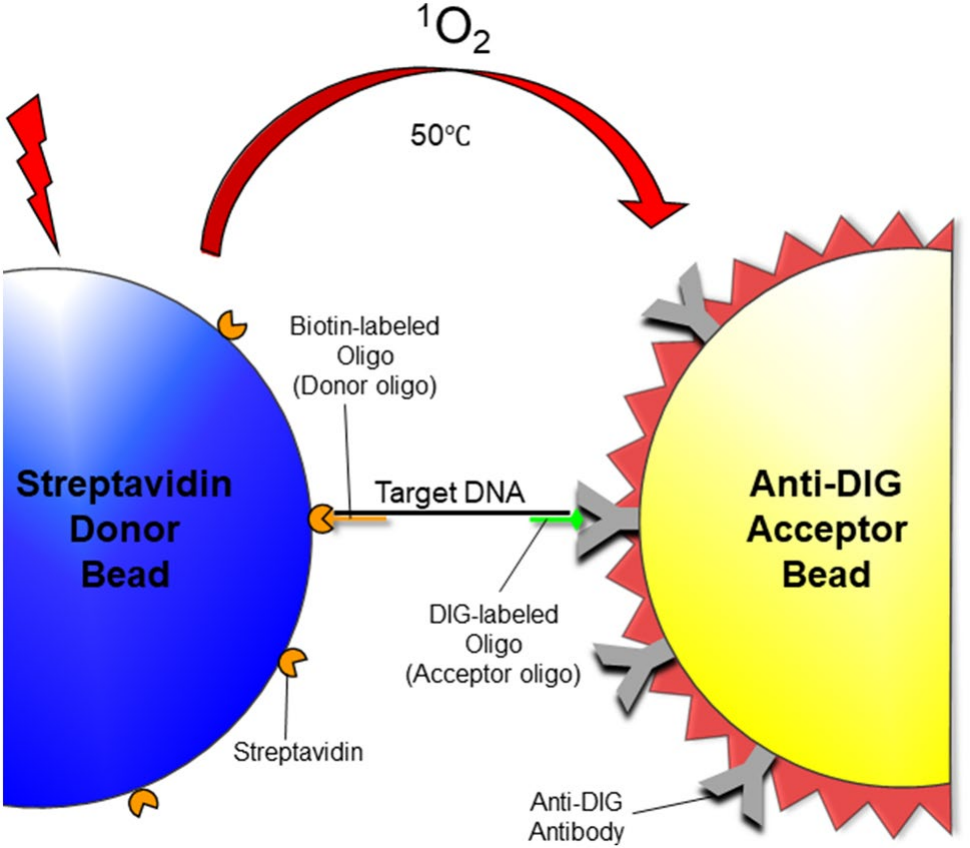
Design of the Oligo-based Amplified Luminescent Proximity Homogenous Assay (oligo-Alpha). Schematic of the detection assay incorporating oligos instead of antibodies for the direct detection of nucleic acid targets. All reactions within the assay as well as the reading of the fluorescent signals were performed at 50 °C.

**Figure 2:**
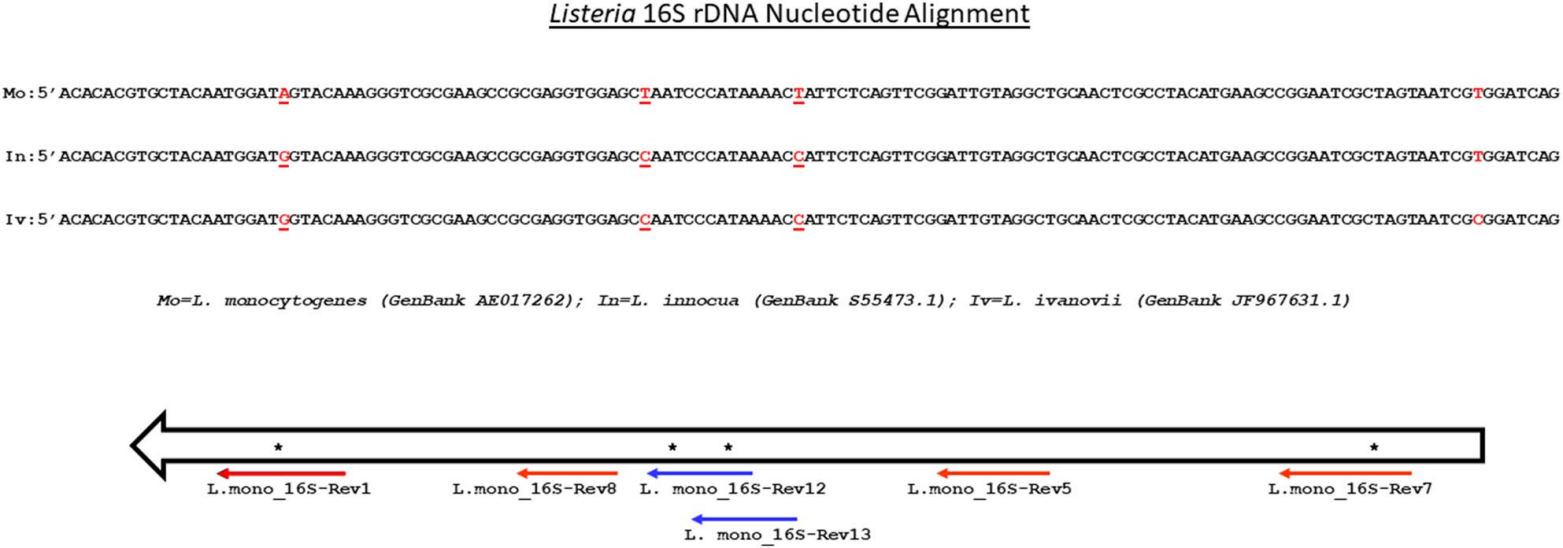
Sequence alignment and primer location. Nucleic acid alignment of the 16S rDNA region for *L. monocytogenes* and the two most closely related species (*L. innocua* and *L. ivanovii*) with nucleotide difference noted in red (top) along with a schematic of the locations of the different oligos used during assay development (bottom). Stars denote nucleotide differences with oligos demarcated by blue lines corresponding to donor oligos and those demarcated by red lines corresponding to acceptor oligos.

The novel oligo-Alpha described herein utilizes the same basic principles as other AlphaLISAs except that instead of conjugating antibodies to both the donor and acceptor beads, oligonucleotides were used as the biorecognition elements (Fig. 1). Using sequence alignments of a 16S rDNA region previously identified for its containment of sequence unique to *L. monocytogenes* compared to other *Listeria* spp.^17,18^, oligos with sequences complementary to those unique regions were synthesized with a dual biotin attached to the 5′ end (Fig. 2 and Supplemental Table 2). The addition of the biotin to the oligo allowed for its conjugation to the corresponding streptavidin-coated donor beads, whereas the presence of the dual biotin specifically helped to stabilize the biotin and protect it from degradation during the sustained incubation period at 50 °C used in the assay^19^. Additional oligos that were complementary to regions within the 16S rDNA were synthesized to contain DIG. The presence of DIG on the oligo allowed for its binding to acceptor beads coated with anti-DIG antibodies. During the initial steps of the assay, the biotin-labeled and the DIG-labeled oligos were incubated statically for 30 min at 50 °C in a buffer containing *Listeria* DNA, which allowed the oligos to bind to their corresponding complementary sequence located on the *Listeria* DNA. Provided that the positioning of the oligos did not overlap with one another, this created a sandwich-style assay with the DNA and the oligos; whereby the target DNA coupled the oligos together in an oligo-DNA-oligo complex. Subsequently, both the donor and acceptor beads were added to the solution and upon binding with their respective oligos (due to the modifications on the oligos) were then brought into close proximity. Therefore, the general process whereby an excited donor bead transmits a signal to an acceptor bead that ultimately emits a detectable fluorescent signal could proceed in the same fashion as AlphaLISAs utilizing antibodies as biorecognition elements. It is worth noting that both the oligo/bead incubation and the reading of the fluorescent signal took place at 50 °C instead of 37 °C, which is typically reported for AlphaLISAs, to increase the binding specificity of the oligos. Initial tests performed by PerkinElmer at this elevated temperature did show a direct correlation between increased temperatures and increased signal outputs, background signals included, but did not appear to have any negative effects on the assay itself. These temperature related changes are likely related to the effects of temperature on the singlet oxygen as has been discussed previously in the literature^20^.

### Optimization of binding conditions

Since oligo binding can be influenced by components such as MgCl_2_ and KCl^21^, various concentrations of MgCl_2_ and KCl were tested in the context of the buffer to identify optimal conditions for binding of the oligos when attached to the corresponding AlphaLISA beads (Supplemental Table 3). Signal intensity of the oligo-Alpha was statistically higher when 4 mM MgCl_2_ was added to the binding buffer compared to 0, 1 mM, 2 mM, 3 mM, and 5 mM concentrations. Comparatively, the signal intensity of the oligo-Alpha was also highest with the addition of 50 mM KCl compared to 0, 25 mM, 75 mM, and 100 mM, although the difference was not statistically significant. In addition, the calculated signal-to-noise ratios were highest at the 4 mM MgCl_2_ and the 50 mM KCl concentrations. Together, these data demonstrated that concentrations of 4 mM MgCl_2_ and 50 mM KCl were optimal for use in this assay and thus were used in the buffer of all subsequent experiments.

### Specific detection of *L. monocytogenes*

**Figure 3:**
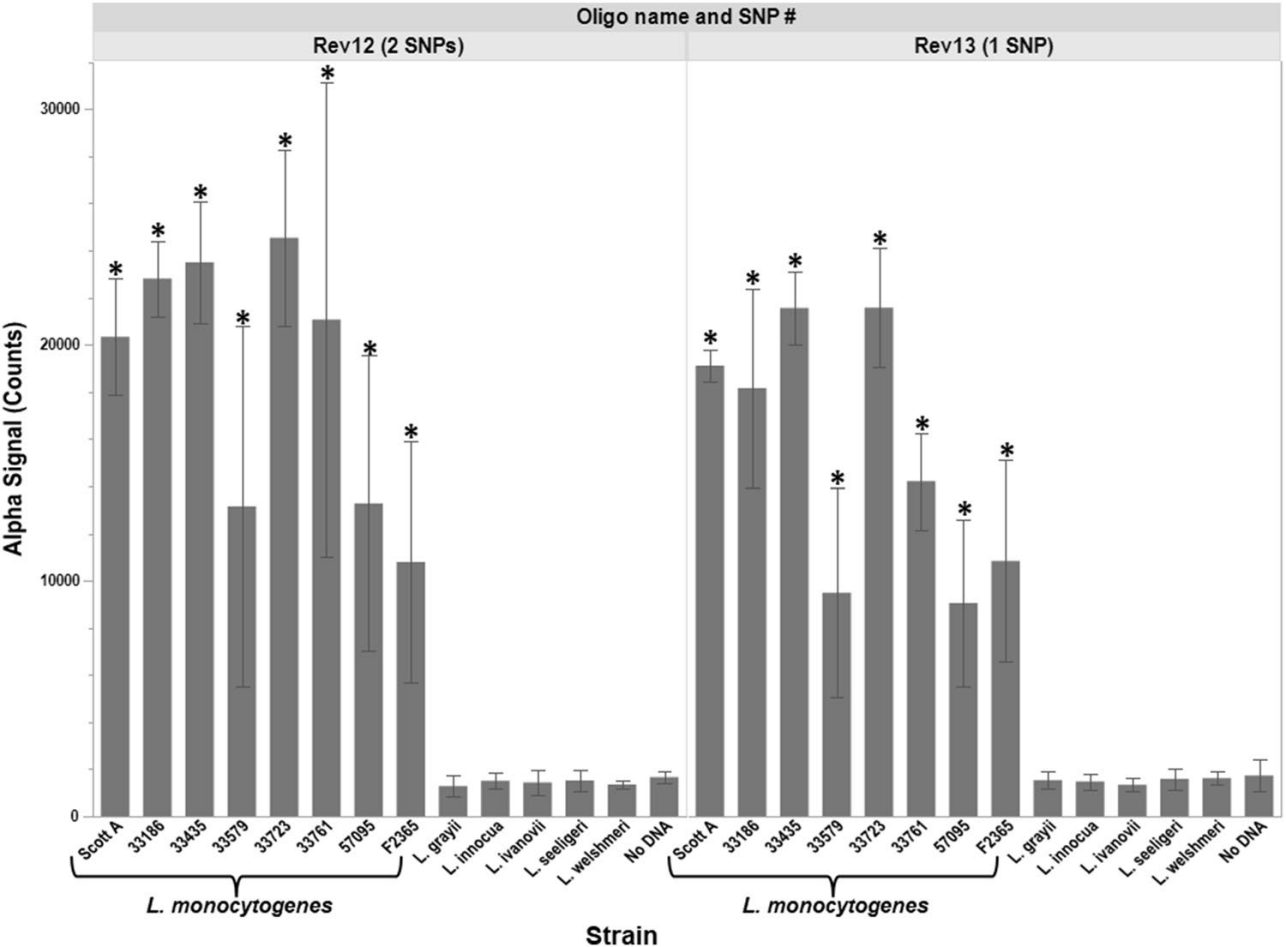
Specificity of oligo-Alpha using oligos with 1 vs 2 nucleotide differences. The ability to specifically detect *L. monocytogenes* was assessed using 90 ng of *Listeria* DNA, a donor oligo containing either 1 SNP (right) or 2 SNPs (left), and the L. mono_16S-Rev7 acceptor oligo. Averages based on four independent trials are shown with signals that are statistically higher (p ≤ 0.05) than the no DNA control denoted by an *. Error bars denote the standard deviation of the mean.

To test the ability of the oligo-Alpha to differentiate *L. monocytogenes* from other *Listeria* species, assays were devised where only the donor oligo contained sequences specific to *L. monocytogenes* (L. mono_16S-Rev12) (Fig. 2 and Supplemental Table 2). The acceptor oligo (L. mono_16S-Rev7) was made to a region that was indistinguishable between *L. monocytogenes* and *L. innocua* (Fig. 2 and Supplemental Table 2). Specificity of the oligo-Alpha using this oligo pair was then tested against 13 different *Listeria* strains, which included six different *Listeria* species (*L. monocytogenes*, *L. innocua*, *L. welshmeri*, *L. seeligeri*, *L. grayii* and *L. ivanovii*) and eight different *L. monocytogenes* isolates encompassing the three most prevalent clinical serotypes (Supplemental Table 1). Results show that signals generated in the presence of *L. monocytogenes* DNA were statistically higher than those generated where either DNA from other *Listeria* spp. were present or contained no DNA within the assay (Fig. 3). Therefore, by attaching oligos to the beads and using temperatures above those typically used for an AlphaLISA, direct detection of *L. monocytogenes* can be achieved. Further testing was performed to determine the ability of the oligo-Alpha to differentiate amongst the *Listeria* spp. when a donor oligo contained only a single nucleotide polymorphism (SNP) compared to a donor oligo containing two SNPs. For this, the oligo L. mono_16S-Rev13 was used in place of L. mono_16S-Rev12 (Fig. 3 and Supplemental Table 2). Results generated determined that the oligo-Alpha could effectively differentiate *L. monocytogenes* from the other *Listeria spp.* based upon the presence of only a single SNP on the donor oligo.

### Detection limits for the oligo-Alpha using a single acceptor

**Figure 4:**
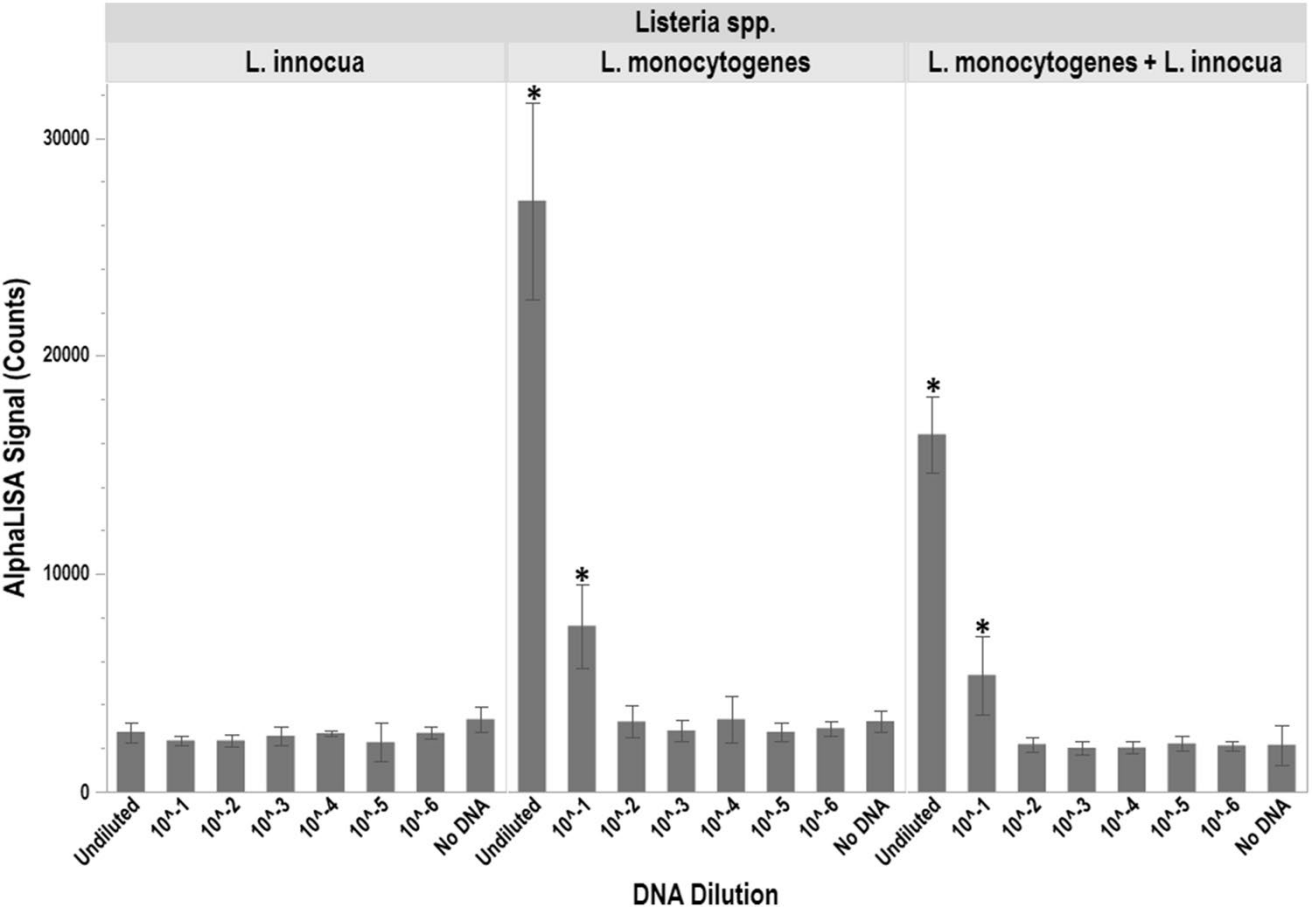
Limit of detection using acceptor oligo L. mono_16S-Rev7. Detection of *L. monocytogenes* by the oligo-Alpha in the presence of homogenous and heterogenous DNA samples. A tenfold serial dilution was performed with initial undiluted DNA amounts as follows: 90 ng of *L. monocytogenes* DNA (left), 90 ng of *L. innocua* DNA (middle), or 90 ng of both *L. monocytogenes* and *L. innocua* DNA (right). Averages based on three independent trials are shown with signals that are statistically higher (p ≤ 0.05) than the no DNA control denoted by an *. Error bars denote the standard deviation of the mean.

The ability to detect *L. monocytogenes* either alone or within a mixed culture using the oligo-Alpha was determined using the donor/acceptor oligo pair, L. mono_16S-Rev12 and L. mono_16S-Rev7, along with DNA fragments from *L. monocytogenes*, *L. innocua* or a combination of *L. monocytogenes* and *L. innocua* DNA fragments (Fig. 4). The DNA was serially diluted from 90 ng (undiluted) down to 90 fg (10^–6^ dilution) and tested in conjunction with the oligo-Alpha to help define the limits of detection for the assay. These data demonstrated the ability of the oligo-Alpha to reliably detect ~ 9 ng of *L. monocytogenes* DNA, even when DNA from another closely related *Listeria* was present in the mixture.

### Enhancing sensitivity through the addition of multiple acceptors

**Figure 5:**
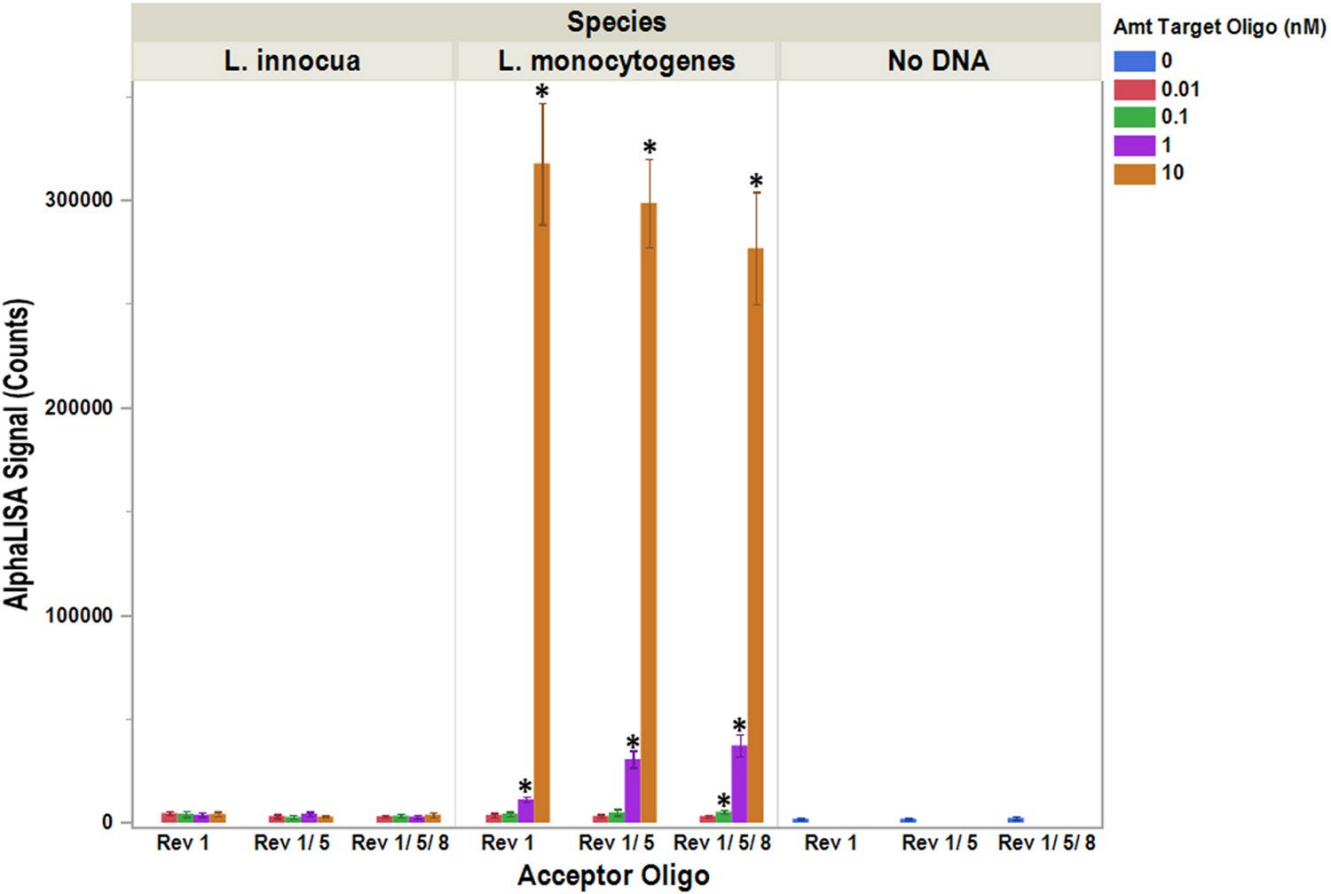
Signal enhancements within assays employing multiple acceptor oligos. Signals produced by assays using 1, 2, or 3 acceptor oligos were recorded for both target (*L. monocytogenes*) and non-target species (*L. innocua*) as well as in the absence of any DNA (no DNA). Assay were performed with various concentrations (0.01 nM, 0.1 nM, 1.0 nM, and 10 nM) of either *L. monocytogenes* or *L. innocua*. Averages based on four independent trials are shown with signals that are statistically higher (p ≤ 0.05) for *L. monocytogenes* compared to *L. innocua* denoted by an *. Error bars denote the standard deviation of the mean.

In theory, the signal emitted by a single donor bead can be received by multiple acceptor beads; whereby increasing the fluorescence emissions and leading to higher assay signals. To determine the effect that multiple acceptor molecules had on the assay’s signal, additional oligos (L. mono_16S-Rev1, L. mono_16S-Rev5, and L. mono_16S-Rev8 in Fig. 1) containing the digoxigenin (DIG) modifications were synthesized. Labeling of multiple oligos with DIG allowed all of them to bind to the anti-DIG antibodies located on the surface of the acceptor beads used in the assay. Care was taken to ensure every oligo was within 70 nucleotides from the location where the donor bead/ oligo resided in an attempt to not exceed the boundaries set forth by the 200 nm diffusion distance for the singlet oxygen. Acceptor oligos (L. mono_16S-Rev1, L. mono_16S-Rev5, and L. mono_16S-Rev8) were then added both separately and in combination to the assay with the results recorded (Fig. 5). From this data, it was determined that when the target concentration was < 10 nM, the highest signal intensity resulted from the use of three acceptor oligos and the lowest signal intensity resulted from the use of a single acceptor oligo. Moreover, the higher signal intensities produced upon the addition of the three acceptor oligos increased the assay’s sensitivity and ultimately allowed 0.1 nM of the *L. monocytogenes* target to be differentiated from the background signal produced when the identical assay was run using *L. innocua.* This tenfold change in the detection limit was only observed with the use of the three acceptor oligos, namely L. mono_16S-Rev1, L. mono_16S-Rev5, and L. mono_16S-Rev8; ultimately achieving a detection limit of 250 amole of target. Interestingly, when the target concentration was high (10 nM), the opposite effect was seen; with the highest signal produced using only a single acceptor oligo (Fig. 5, orange bars). Despite this discrepancy, accurate detection of *L. monocytogenes* was still obtained at target concentration of 10 nM from assays employing three acceptor oligos since the signals produced were well above those seen with either no DNA or the *L. innocua* controls*.*

### Detection of *L. monocytogenes* within food matrices

**Figure 6:**
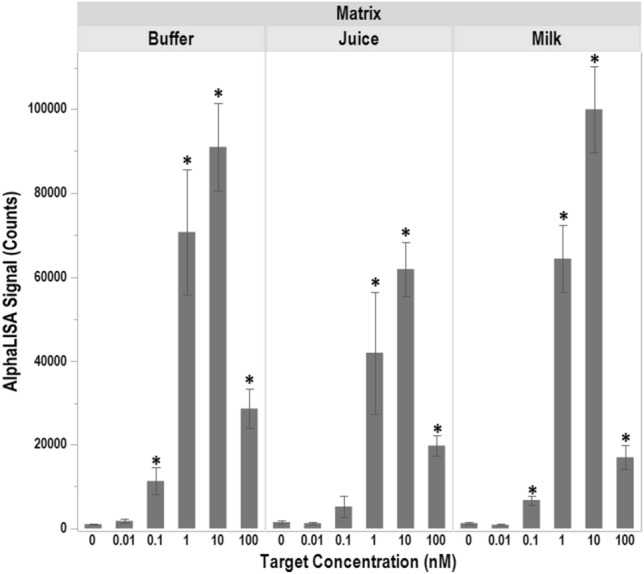
Use of the oligo-Alpha for the detection of *L. monocytogenes* in food matrices. Apple juice and non-fat milk were tested in conjunction with the oligo-Alpha to determine its ability to detect *L. monocytogenes* within food matrices. Buffer was used as a control. Averages based on three independent trials are shown with signals that are statistically above those recorded for the 0 nM concentrations (p ≤ 0.05) denoted by an *. Error bars denote the standard deviation of the mean.

*Listeria monocytogenes* has been isolated from numerous contaminated retail products including milk and apple juice^22,23^ with Hazard Analysis and Critical Control Point (HACCP) procedures being set in place by the Food and Drug Administration for both products in an effort to limit foodborne illness associated with their consumption. Therefore, the ability of the oligo-Alpha to detect *L. monocytogenes* within these matrices was also investigated. For this, side-by-side comparisons were made between assays performed in buffer, apple juice, and non-fat milk, to determine if *L. monocytogenes* was detectable within these matrices (Fig. 6). Various target concentrations (0.01, 0.1, 1, 10, and 100 nM) of a single-stranded 16S rDNA fragment from *L. monocytogenes* was added in conjunction with either buffer, apple juice, or milk and the resulting oligo-Alpha signals were recorded. Samples that did not contain DNA were used as controls. Data collected shows that the signals generated from assays containing ≥ 1 nM of target DNA were statistically higher than those generated in the absence of DNA for all of the matrices tested, indicating that *L. monocytogenes* could be detected in both milk and apple juice using the oligo-Alpha. Additionally, signals generated in either buffer or milk at target concentrations of 0.1 nM were statistically increased above background whereas those generated in apple juice were not despite the mean signal intensity being higher than that of the control (p = 0.0072, 0.0162, and 0.2467 for buffer, milk, and apple juice respectively). For all of the assays conducted using 0.01 nM of target, none were found to be significantly above the background no DNA control.

### Application of the oligo-Alpha to crude cell lysates

**Figure 7:**
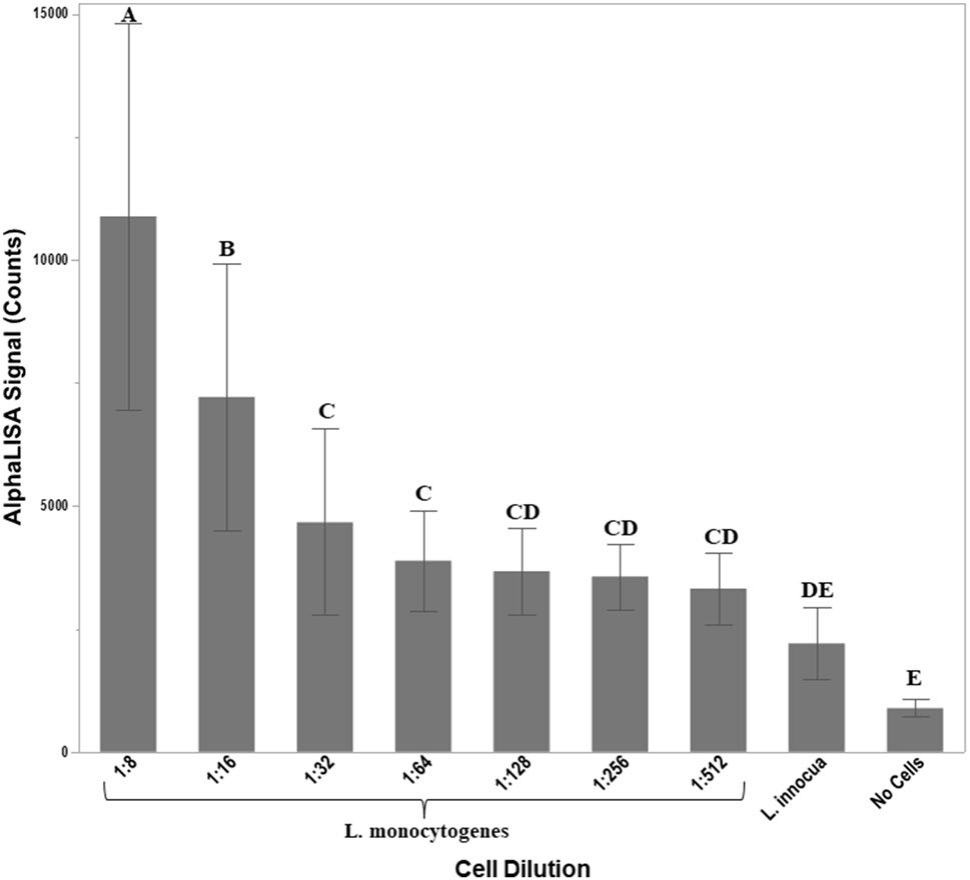
Identification of *L. monocytogenes* from whole cell lysates via the oligo-Alpha. The applicability of the oligo-Alpha for detecting pathogenic cells was tested by examining the ability of the oligo-Alpha to differentiate *L. monocytogenes* from *L. innocua* using a series of diluted crude cell lysates. The OmniLyse cell disruptor was used to thoroughly lyse log phase cultures for testing. Averages from three independent trials are shown with error bars denoting the standard deviation of the mean. Tukey’s honestly significant difference test defined statistical differences amongst signals and are denoted by dissimilar letters.

To determine if the oligo-Alpha could identify and differentiate *L. monocytogenes* using crude cell lysates, tests were conducted on both *L. monocytogenes* and *L. innocua* grown in culture to log phase. Bacterial loads were enumerated using the 6X6 drop plate method with the number of live bacteria within each mL of cultured cells ranging from 688 to 826 million for *L. monocytogenes* and 1.2 billion to 1.8 billion for *L. innocua* (Supplemental Table 4). Crude cell lysates were also obtained from the culture by homogenizing the cells with the OmniLyse cell disruptor. Using a 1:2 dilution ratio, lysates were serial diluted and seven different dilutions (1:8, 1:16, 1:32, 1:64, 1:128, 1:256, and 1:5:12) were analyzed via the oligo-Alpha (Fig. 7). Results demonstrated a higher signal intensity with all diluted lysates of *L. monocytogenes* compared to the no cell control. *L. monocytogenes* was also able to be differentiated from *L. innocua* within whole cell lysates diluted to 1:64. Based upon the 6 × 6 drop plate enumeration method, the approximate number of cells present within the well at this dilution was calculated to be 1.17 × 10^5^ (Supplemental Table 4).

## Discussion

Differentiation of *L. monocytogenes* from other *Listeria* species is important in the context of preventing foodborne illness since only *L. monocytogenes* is consistently associated with human disease^24^. Unfortunately, detection of *L. monocytogenes* is not only time-consuming, but the methods are complex with atypical *Listeria* spp. being a source of interpretation error when using certain protocols. For example, phenotypic tests utilized by the Food Safety and Inspection Service (FSIS) cannot always distinguish *L. monocytogenes* from *L. innocua,* which can result in an incorrect classification of the bacterium present^25^. This suggests that methods based on genetic differentiation may be more reliable for this pathogen, and thus, *L. monocytogenes* was chosen as a priority target for the production of an oligo-Alpha detection platform.

In general, the use of DNA targets can overcome several limitations of protein-based methods. Unlike protein markers, DNA markers that can distinguish a target of interest are readily available for most targets. Furthermore, detection methods utilizing proteins rely upon the stable expression of markers during the detection process and often times require the protein to be maintained within a certain conformation. This can be difficult to achieve considering protein markers can be modified by enzymes within a sample. Also, protein markers located on the surface of cells may be prone to cleavage by shear forces applied during sample collection and/or sample processing. Moreover, the solubility of proteins can vary in aqueous solutions whereas DNA is readily soluble in water.

As with other AlphaLISAs developed, the method presented here can be readily integrated into high-throughput workflows since it was conducted in a high-density screening format using an automatable multimode plate reader that can be equipped with a plate stacker. It may be possible to further simplify the assay by releasing the DNA from cells using heat as a mode for lysis. Although not tested here due to heating limitations of the plate reader employed, it is likely this could be performed in a single assay plate since temperature-sensitive enzymes are not required for the oligo-Alpha. Doing so would allow colonies or culture enrichments to be tested in an extremely simple and rapid fashion. In addition, certain design aspects such as using RNA as the target instead of DNA may further enhance detection of a particular target as has been seen in other assays^26,27^.

Differentiating pathogenic from non-pathogenic species is vital to the development of a detection assay. In PCR, the use of two non-overlapping oligo probes that are specific to only the target of interest are typically employed to help increase the specificity of the assay and decrease the rate of false-positive reactions. However, for some targets, multiple regions that are unique to the target of interest either may not exist or may not exist within a distance suitable for PCR. Unfortunately, PCR applications that utilize a single unique primer in conjunction with a common primer are less reliable compared to PCR assays that use two unique oligos because of the higher rate of non-specific/off-target amplification that occurs^28^. Because the oligo-Alpha presented here was able to effectively differentiate *L. monocytogenes* from the other *Listeria spp.* based upon the presence of a single SNP within the donor oligo, it may be possible to use the oligo-Alpha under circumstances where PCR fails to reliable distinguish amongst targets.

During this study, it was observed that the signals generated by the oligo-Alpha when both *L. monocytogenes* and *L. innocua* were present in a single sample appeared slightly lower than those generated in the presence of *L. monocytogenes* alone (Fig. 4). This was likely due to the fact that the acceptor L. mono_16S-Rev7 oligo used had 100% sequence identity to both *L. monocytogenes* and *L. innocua* (Fig. 1), thereby limiting the reagent’s availability in the assay. The generation of lower signals may also be compounded by the presence of twice the amount of nucleic acid material (ex: 90 ng from the target *L. monocytogenes* and 90 ng from the non-target *L. innocua*) in the assay, which can decrease the number of interactions between the oligos and their intended targets. Optimization of oligo amount may help alleviate some of these factors.

In addition, it was noted that the mean signal intensity for the assays involving apple juice were typically lower than that for milk and buffer. It is suspected that differences in pH may have attributed to these lower signals. Upon measuring the pH, these matrices were noted to be vastly different with apple juice having a pH of ~ 4, milk ~ 7, and buffer ~ 8. Not only could the lower pH affect the ability of the oligo to bind to the target, but it could also affect the lifetime of the singlet oxygen^29^. As expected, the applicability of the oligo-Alpha would need to be tested for each matrix prior to broad implementation.

Key to our ability to obtain an increased signal and corresponding lower limit of detection when additional acceptors are added to the oligo-Alpha is the fact that each donor bead is estimated to be able to emit up to 60,000 single oxygen molecules per second upon illumination^2^. This allows a single donor to activate multiple acceptor beads present within ~ 200 nm of the donor, a distance that is set by the limit of diffusion during the 4 microsecond half-life of the singlet oxygen^2^. Activation of multiple acceptor beads increases the emitted fluorescence and results in higher assay signals. Although the increase in signal intensity observed was not directly additive when two or three acceptor oligos were used, it was higher than the results observed with a single oligo (Fig. 5). Generally, assays containing three acceptor oligos yielded higher signals than those containing two acceptors, which in-turn yielded a higher signal than assays containing a single oligo. This increase in signal can ultimately improve the sensitivity of an assay when low levels of target are present and suggest that the limit of detection for any given oligo-Alpha can be modified by simply adding/removing acceptor-binding oligo sequences. Future studies will help define the saturation point where additional oligos no longer result in an increased signal.

At first glance, detection limits for the oligo-Alpha may appear to vary depending upon the type of nucleic acid detected (e.g. oligos versus cell lysates) (Figs. 5 and 7). However, when one considers that crude cell lysates allow cellular RNA as well as DNA to serve as a target and the fact that the number of ribosomes (which are composed primarily of rRNA) within a single *L. monocytogenes* cell can range from 25,000 during exponential growth to 600 during stationary phase^30^, the values obtained are relatively similar for the assays performed (Table 1). Although calculations defining the copy number for the targets within each assay at the lowest detectable level demonstrated that more targets were needed when DNA fragments were utilized compared to the other assays, this is likely a result of the smaller number of acceptors used with this assay as discussed above.

**Table 1:** Copy number calculations for assays performed.

	DNA fragment	Oligo fragment	*L. monocytogenes* cells
Associated figure	4	5	7
No. of acceptor oligos used	1	3	3
Length (bases)	938	195	-
Oligo-Alpha detection limit	9 ng	0.1 nM	1.17E + 05 cells
Nucleic acid strands	Double	Single	Double
Copy number at detection limit	9.4E+09	1.5E+08	7.0E+08 to 2.9E+09*

*The number of ribosomes within a single *L. monocytogenes* cell has been reported to range from between 25,000 during exponential growth to 600 during stationary phase^30^.

When designing oligos for use in an oligo-Alpha, there are several factors that should be considered. For example, oligos spacing is crucial because not only can the acceptor(s) be placed too far from the donor to receive the emitted signal but arrangements where oligos are too close may also be detrimental because steric hinderance could prevent both beads from being able to bind. The helical structure of DNA is also a factor that should be considered when determining the proper distances. The predominant form of DNA, B-DNA, is a double helix that is ~ 2 nm wide with a distance between base pairs of 0.34 nm^31^. Given its pitch of 3.4 nm (i.e., the helix completes a turn every ~ 10 base pairs), oligo distances may be closer together or further apart than if the molecule were simply linear. Future studies are needed to identify the effect of oligo length, G+C content, and assay temperature on signal intensity. Additional design factors include SNP placement. As reported here, the SNP(s) were incorporated into the donor oligo since it is the donor bead that has the ability to activate multiple acceptors. Had the donor oligo not contained the SNP(s), each acceptor would need to be made to a region that allowed it to be differentiated from the other *Listeria* species; a situation far from ideal when employing multiple acceptors.

In summary, replacement of antibodies with oligonucleotides in this assay has broad applicability and greatly expands the types of targets identifiable via the Alpha technology because it provides the opportunity to make use of both phenotypic as well as genotypic differences. It may also aid in driving down assay costs and production times since oligonucleotide synthesis is often more rapid and inexpensive than antibody production. Given that newly emerging pathogens can now be sequenced without isolation, direct detection of nucleic acids by the oligo-Alpha may facilitate its implementation as a method to detect novel targets, including viable but non-culturable organisms and those lacking high-quality antibodies. Although established here for the detection of *L. monocytogenes*, one of the most significant features of the oligo-Alpha is its ability to differentiate targets based upon only a single nucleotide difference. The desire to detect SNPs expands far beyond pathogen detection, especially where human health is concerned. SNPs have been used as biological markers for the prediction of certain aspects of patient care such as their response to a particular drug, an individual’s susceptibility to certain environmental factors, and their overall risk of developing disease such as heart disease, diabetes, and cancer^32^. Hence, expansion of the AlphaLISA technology into these additional diagnostic markets is likely to be achieved in the near future.

### Supplementary Information

Supplementary Tables.

## Data Availability

All datasets used and/or analyzed during the current study are available from the corresponding author upon reasonable request.
