# Supplementary material for: High-throughput homogenous assay for the direct detection of Listeria monocytogenes DNA
Source: Sci Rep. 2024 Mar 25;14:7026. doi: 10.1038/s41598-024-56911-8 (PMC10963731; doi:10.1038/s41598-024-56911-8)
Supplement: Supplementary file 1 — Supplementary Tables. [file 41598_2024_56911_MOESM1_ESM.docx]

**High-throughput homogenous assay for the direct detection of *Listeria monocytogenes* DNA**

Cheryl M. Armstrong*, Joseph A. Capobianco, Sarah Nguyen, Manita Guragain, and Yanhong Liu

**Supplemental Tables:**

**Supplemental Table 1: *Listeria* strains utilized within the present study.**

| Name | Serotype | Isolation Information |
| --- | --- | --- |
| *L. grayii* 700545 | N/A | ATCC700545 |
| *L. innocua* | N/A | ATCC51742 |
| *L. ivanovii* | N/A | ATCC49954 (food isolate) |
| *L. monocytogenes* 33186 | 1/2b | 20674-01 |
| *L. monocytogenes* 33435 | 1/2a | FSIS isolate |
| *L. monocytogenes* 33579 | 1/2c | J.2315 |
| *L. monocytogenes* 33723 | 1/2a | FSIS isolate |
| *L. monocytogenes* 33761 | 1/2c | FSIS isolate |
| *L. monocytogenes* 57095 | 1/2b | FSIS isolate |
| *L. monocytogenes* F2365 | 4b | Isolated from Mexican style cheese |
| *L. monocytogenes* Scott A | 4b | Human isolate |
| *L. seeligeri* 35967 | N/A | ATCC35967 |
| *L. welshmeri* 35897 | N/A | ATCC35897 |

**Supplemental Table 2**: **Primer and single stranded DNA fragments used for the development of the oligo-Alpha.**

| Target and primer/oligo name | Sequence | Oligo Modification | Reference |
| --- | --- | --- | --- |
| **Oligo attached to Alpha beads** |  |  |  |
| L. mono_16S-Rev1 | Ctatccattgtagcacgtgtg | 5’ DIG | This study |
| L. mono_16S-Rev5 | GCAGCCTACAATCCGAACTG | 5’ DIG | This study |
| L. mono_16S-Rev7 | Ctgatccacgattactagcgat | 5’ DIG | This study |
| L. mono_16S-Rev8 | Ctgatccacgattactagcgat | 5’ DIG | This study |
| L. mono_16S-Rev12 | GAATAGTTTTATGGGATTAGCT | 5’ biotin | This study |
| L. mono_16S-Rev13 | CTGAGAATAGTTTTATGGGATT | 5’ biotin | This study |
|  |  |  |  |
| **16S rDNA amplification primer** |  |  |  |
| U1 | CAGCMGCCGCGGTAATWC | - | [15] |
| LI1 | CTCCATAAAGGTGACCCT | - | [15] |
|  |  |  |  |
| **16S rDNA target oligo** |  |  |  |
| L. mono_16S-Seq (1193-1387) | GACGTCAAATCATCATGCCCCTT ATGACCTGGGCTACACACGTGC TACAATGGATAGTACAAAGGGT CGCGAAGCCGCGAGGTGGAGC TAATCCCATAAAACTATTCTCA GTTCGGATTGTAGGCTGCAACT CGCCTACATGAAGCCGGAATCG CTAGTAATCGTGGATCAGCATG CCACGGTGAATAC GTTCCC | - | GenBank ID AE01726 |
|  |  |  |  |
| L. innocua_16S-Seq (1134-1328) | GACGTCAAATCATCATGCCCCT TATGACCTGGGCTACACACGTG CTACAATGGATGGTACAAAGGG TCGCGAAGCCGCGAGGTGGAGC CAATCCCATAAAACCATTCTCAG TTCGGATTGTAGGCTGCAACTCG CCTACATGAAGCCGGAATCGCTA GTAATCGTGGATCAGCATGCCAC GGTGAATACGTTCCC | - | GenBank ID S55473.1 |
|  |  |  |  |

**Supplemental Table 3: MgCl_2_ and KCl optimization trials.**

| Test | Compound | | Alpha Signal^+^ | | | Signal/  Noise |
| --- | --- | --- | --- | --- | --- | --- |
|  | [MgCl_2_]  (mM) | [KCl]  (mM) | *L. monocytogenes^++^* | *L. innocua* | Negative Control |  |
| MgCl_2_  Test | 0 | 50 | 3741^c^ | 2877 | 2960 | 1.26 |
|  | 1 | 50 | 7003^c^ | 2444 | 2796 | 2.50 |
|  | 2 | 50 | 15097^c^ | 2317 | 2790 | 5.41 |
|  | 3 | 50 | 23120^bc^ | 2136 | 2392 | 9.67 |
|  | 4 | 50 | 56990^a^ | 2746 | 2686 | 21.22 |
|  | 5 | 50 | 43952^ab^ | 2016 | 2484 | 17.69 |
| KCl  Test | 4 | 0 | 56990^*^ | 2746 | 2686 | 21.22 |
|  | 4 | 25 | 55709^*^ | 2601 | 3247 | 17.16 |
|  | 4 | 50 | 68670^*^ | 2541 | 2315 | 29.67 |
|  | 4 | 75 | 51081^*^ | 2013 | 2162 | 23.63 |
|  | 4 | 100 | 58844^*^ | 2074 | 2144 | 27.45 |

^+^Mean values from 3 independent trials. ^++^Significance between Alpha signal values are denoted by dissimilar letters (top) or as an * (bottom) as determined by a Student’s t-test at a 95% confidence level.

**Supplemental Table 4: Cell counts based upon the 6x6 drop plate method.**

|  | *L. monocytogenes* | | | | *L. innocua* | | | |
| --- | --- | --- | --- | --- | --- | --- | --- | --- |
|  | Trial 1 | Trial 2 | Trial 3 | Average | Trial 1 | Trial 2 | Trial 3 | Average |
| Plate counts (CFU/mL) | 7.34E+08 | 6.88E+08 | 8.26E+08 | 7.49E+08 | 1.40E+09 | 1.17E+09 | 1.83E+09 | 1.06E+09 |
| Cells/Well (undiluted) | 7.34E+06 | 6.88E+06 | 8.26E+06 | 7.49E+06 | 1.40E+07 | 1.17E+07 | 1.83E+07 | 1.06E+07 |
| Cells/Well (1:2 dilution) | 3.67E+06 | 3.44E+06 | 4.13E+06 | 3.75E+06 | 7.00E+06 | 5.85E+06 | 9.15E+06 | 5.28E+06 |
| Cells/Well (1:4 dilution) | 1.84E+06 | 1.72E+06 | 2.07E+06 | 1.87E+06 | 3.50E+06 | 2.93E+06 | 4.58E+06 | 2.64E+06 |
| Cells/Well (1:8 dilution) | 9.18E+05 | 8.60E+05 | 1.03E+06 | 9.37E+05 | 1.75E+06 | 1.46E+06 | 2.29E+06 | 1.32E+06 |
| Cells/Well (1:16 dilution) | 4.59E+05 | 4.30E+05 | 5.16E+05 | 4.68E+05 | 8.75E+05 | 7.31E+05 | 1.14E+06 | 6.60E+05 |
| Cells/Well (1:32 dilution) | 2.29E+05 | 2.15E+05 | 2.58E+05 | 2.34E+05 | 4.38E+05 | 3.66E+05 | 5.72E+05 | 3.30E+05 |
| Cells/Well (1:64 dilution) | 1.15E+05 | 1.08E+05 | 1.29E+05 | 1.17E+05 | 2.19E+05 | 1.83E+05 | 2.86E+05 | 1.65E+05 |
| Cells/Well (1:128 dilution) | 5.73E+04 | 5.38E+04 | 6.45E+04 | 5.85E+04 | 1.09E+05 | 9.14E+04 | 1.43E+05 | 8.26E+04 |
| Cells/Well (1:256 dilution) | 2.87E+04 | 2.69E+04 | 3.23E+04 | 2.93E+04 | 5.47E+04 | 4.57E+04 | 7.15E+04 | 4.13E+04 |
| Cells/Well (1:512 dilution) | 1.43E+04 | 1.34E+04 | 1.61E+04 | 1.46E+04 | 2.73E+04 | 2.29E+04 | 3.57E+04 | 2.06E+04 |
